# The Thoracic Footprint of Hepatocellular Carcinoma: A Mechanism-Based Imaging Narrative Review

**DOI:** 10.3390/diagnostics16193072

**Published:** 2026-09-22

**Authors:** Carmen Georgiana Pitusac Prisacaru, Ramona Cadar, Cristian Dumitru Lupascu, David Toma, Adriana Loredana Pintilie, Tudor Birladeanu, Ruxandra Stirbu, Raluca Teodora Tiron, Roxana Cobzariu, Radu Crisan Dabija, Corina Lupascu Ursulescu

**Affiliations:** 1 “Grigore T. Popa” University of Medicine and Pharmacy, 700115 Iasi, Romania; prisacaru.georgiana@yahoo.com (C.G.P.P.); tibaramona@yahoo.com (R.C.); cristian_lupascu@yahoo.com (C.D.L.); tomadavidro@gmail.com (D.T.); birladeanu.tudor@yahoo.com (T.B.); radu.dabija@umfiasi.ro (R.C.D.); corina.ursulescu@gmail.com (C.L.U.); 2 Department of Radiology and Medical Imaging, Clinical Hospital of Pneumophthisiology, 700115 Iasi, Romania; 3 Department of Surgery, “Sf. Spiridon” Clinical Emergency Hospital, 700116 Iasi, Romania; 4 Pulmonology Clinic, Clinical Hospital of Pneumophthisiology, 700115 Iasi, Romania; rux.stirbu@gmail.com (R.S.); tironraluca@yahoo.com (R.T.T.); cobzariuroxana6@gmail.com (R.C.); 5 Department of Radiology and Medical Imaging, “Sf. Spiridon” Clinical Emergency Hospital, 700116 Iasi, Romania

**Keywords:** hepatocellular carcinoma, thoracic imaging, pulmonary metastases, tumour thrombus, pleural disease, treatment complications, narrative review

## Abstract

A thoracic finding in a patient with hepatocellular carcinoma (HCC) may represent metastatic disease, vascular extension, treatment-related injury, or a complication of cirrhosis, and each carries different implications for staging and care. The mechanism behind the finding therefore matters as much as the finding itself. We searched the PubMed/MEDLINE database for articles published up to August 2026 and screened the reference lists of retrieved articles for additional relevant publications. The narrative review is organised into four domains: direct oncological involvement, vascular dissemination, treatment-related injury, and cirrhosis-associated mimics. Within each domain, computed tomography (CT) provides most of the chest assessment, while magnetic resonance imaging, echocardiography, and angiography address more specific anatomical or diagnostic questions. We next discuss common patterns of pulmonary metastasis alongside benign nodules common in transplant candidates, and the biological and small-lesion limitations of functional imaging. Thoracic findings gain meaning in the context of hepatic disease, vascular anatomy, and treatment history. Enhancement and alpha-fetoprotein have never been validated as noninvasive rule-out tests for pulmonary metastasis, so the choice between sampling and surveillance rests on multidisciplinary judgement of the costs of uncertainty, procedural feasibility, and evidence strength.

## 1. Introduction

Primary liver cancer caused an estimated 759,000 deaths worldwide in 2022, and hepatocellular carcinoma (HCC) accounts for most primary hepatic malignancies [1]. The thorax is relevant to HCC for two opposing reasons. Extrahepatic spread alters staging and usually shifts treatment towards systemic therapy; conversely, a benign pulmonary nodule or a cirrhosis-related complication, once misread as metastatic disease, can deprive a patient of a curative option [2,3,4]. In a patient being assessed for transplantation or resection, the way this uncertainty is resolved therefore determines whether either procedure remains available [5].

Pulmonary metastases are the most common thoracic manifestation of extrahepatic HCC, but they represent only part of what the chest can show [6,7,8]. A tumour at the hepatic dome may grow through the diaphragm; a tumour within a hepatic vein may extend into the inferior vena cava (IVC) and the right atrium, and, from there, the pulmonary circulation; and treatment delivered via extrahepatic arterial collaterals may injure the pleura or the adjacent lung [9,10,11,12,13]. Airway, pleural, pericardial and chest-wall lesions are documented far less well and may present with haemoptysis, an effusion or another respiratory symptom that first brings the patient to the respiratory physician [14,15,16,17,18].

Earlier radiographic descriptions, and the reviews that followed them, mapped the range of thoracic and extrahepatic HCC findings [16,19,20]. This review places those observations alongside the complications of contemporary treatment and the diagnostic procedures that fall to the respiratory physician. The discussion is organised in four domains, namely, direct oncological involvement, vascular dissemination, treatment-related injury and cirrhosis-associated mimics (Figure 1), with extrahepatic collateral arterial supply treated within the domain of locoregional therapy, where its consequences arise. We aim to add a practical reading of thoracic findings in the context of hepatic disease, vascular anatomy, and treatment history, including the most challenging case: an indeterminate nodule found before curative treatment.

However, the evidence supporting these manifestations is uneven, and this unevenness has shaped how the review is written. Cohorts and dedicated imaging studies support pulmonary metastases and macrovascular invasion, whereas endobronchial, pleural, pericardial, and pulmonary microvascular manifestations rest largely on single cases or small series [7,9,14,17,18,21,22]. We keep this difference visible in the text and in Table 1 because a manifestation reported once establishes an anatomical possibility, while its frequency and imaging specificity can only be derived from cohorts.

## 2. Materials and Methods

We searched the PubMed/MEDLINE database for articles published up to 1 August 2026, combining the term “hepatocellular carcinoma” with terms for the thoracic sites and manifestations of interest (pulmonary metastasis, endobronchial disease, pleura, diaphragm, pericardium, venous tumour thrombus, pulmonary tumour embolism) and with imaging terms (computed tomography (CT), magnetic resonance imaging (MRI), tomography, diffusion). Separate searches addressed inferior phrenic arterial treatment, radioembolisation, ablation-related fistulas and immune-related pneumonitis. We screened the reference lists of the retrieved articles for additional relevant publications and ran supplementary searches by article title, author, and digital object identifier (DOI) through Europe PMC. All references cited in this review were subsequently verified against the original publications. Bibliographic details were checked against indexed records, Crossref metadata, and publishers’ pages, and the quantitative and diagnostic claims attributed to each study were confirmed in the original report or in an accessible full text; this verification was completed in September 2026.

Selection was purposive rather than exhaustive, and studies were retained according to their relevance to thoracic imaging in HCC. We preferred HCC-specific cohorts and imaging series, and used guidelines and systematic reviews to provide context; where no HCC-specific study was available, we used evidence from the general thoracic literature and identified it as such in the text. We retained case reports because, for several rarer manifestations, they represent the only published documentation. We preferred English-language publications, although we included one older Japanese surgical report based on its English abstract, and we did not use conference educational material as a source of evidence. Because the search was targeted rather than systematic, a single reviewer screened the results; we described the strength of the evidence qualitatively alongside each finding rather than formally scoring it, and we synthesised the data narratively without pooling. Reporting follows the principles of the Scale for the Assessment of Narrative Review Articles (SANRA), with study populations and limitations stated next to the findings [23]. Figure 1 presents four major directions for interpreting thoracic findings in HCC.

## 3. Pulmonary Parenchymal and Airway Metastases

### 3.1. Clinical Context and Common CT Finding

The probability of pulmonary metastasis varies considerably by the study population. In a registry analysis of 33,177 patients with HCC, 2084 (6.28%) had pulmonary metastases at initial diagnosis [6]. In contrast, among 172 selected liver-transplant candidates, 76.2% had at least one pulmonary nodule, yet only 2.7% of the 301 nodules were classified as HCC metastases, 1.0% as another malignancy and 2.7% as infection [5]. However, both proportions were calculated per nodule in a population already selected for limited hepatic disease, so a newly diagnosed patient with a large tumour and macrovascular invasion falls into a considerably different pre-test range. Dissemination may occur synchronously or metachronously; the relative frequency of the two patterns remains uncertain, because the series reporting timing were assembled for metastasectomy and therefore describe patients selected for surgery rather than the disease as a whole [6,24].

Larger or multiple intrahepatic tumours, macrovascular invasion, and a raised alpha-fetoprotein (AFP) all increase concern for extrahepatic disease, although none can account for an individual lung nodule, which must be judged on its own imaging features [6,19,25,26]. Similarly, vascular invasion facilitates haematogenous dissemination, but pulmonary metastasis is also encountered in patients whose imaging shows no vascular invasion. Therefore, interpret the current hepatic examination alongside prior liver-directed treatment and the duration of intrahepatic disease control [19,20,24].

On CT, pulmonary HCC metastases usually appear as solid, round or lobulated nodules of varying size, often multiple and bilateral, distributed randomly, peripherally or at the lung bases [19,20,27]. In a cohort of 76 patients with pulmonary metastases and no other metastatic site, for example, 59 (77.6%) had multiple nodules, and 35 (46.1%) had bilateral disease [27], although a solitary lesion remains possible. Enlargement over time, or the appearance of new nodules, increases suspicion, with the reservation that infection or another malignancy may grow in the same way. A feeding vessel or a haemorrhagic halo may accompany a metastasis; however, both signs are described in the wider literature on pulmonary metastases and are shared with deposits from other primary tumours [28,29].

HCC may remain hypervascular in its pulmonary deposits. A retrospective early-phase contrast CT study comparing 13 patients with pulmonary HCC metastases with 25 patients with primary lung cancer found greater enhancement in the metastatic group [30]. Given the small sample, the single early acquisition, and the choice of primary lung cancer as comparator, this finding describes a difference between two malignancies. A washout criterion for the lung, or an enhancement-plus-AFP rule that could stand in for tissue confirmation, would require a benign-nodule comparator and a validation cohort, neither of which is currently available. In addition, many staging chest CT examinations are acquired without intravenous contrast, which further limits the routine use of enhancement [2].

Fluorodeoxyglucose (FDG) avidity reflects the biology of HCC, so a well-differentiated tumour may take up very little tracer [31]. In addition, positron emission tomography/computed tomography (PET/CT) has limited sensitivity for small pulmonary lesions, particularly those below approximately 8 mm, because of spatial resolution, partial-volume effects and respiratory motion [29,31,32]; this threshold varies with the scanner, reconstruction and tracer uptake, and a nodule below it simply lies beyond what PET/CT can assess. Consequently, a negative FDG study does not exclude a small pulmonary HCC metastasis, and thin-section CT remains the principal examination for detecting nodules and comparing them over time.

### 3.2. An Indeterminate Nodule Before Curative Treatment

When a solitary 6 mm nodule is detected during pretransplant staging, the first step is to compare it with any prior thin-section CT, since morphology, the pattern of calcification, and the duration of documented stability may together support a benign explanation; without such evidence, the nodule remains indeterminate (Figure 2). In the transplant cohort cited in Section 3.1, none of the malignant nodules was triangular or lentiform in shape or calcified [5]; these features therefore favour benignity in this population, within the limits of a single retrospective series. Stability at a single short-interval examination, on the other hand, is insufficient to classify a nodule as benign.

The extent of the investigation depends on whether the answer would change management. Tissue sampling becomes reasonable for a new or enlarging nodule when the result would affect eligibility for curative treatment, or the treatment of an infection or a second primary cancer [5]. At 6 mm, however, sampling may be technically difficult, and its expected yield has to be weighed against the nodule’s location, bleeding risk, and the patient’s general condition. When immediate sampling is unlikely to settle the question, an individualised CT reassessment after multidisciplinary discussion represents a defensible alternative. We use an interval of about three months as a working example, while acknowledging that urgency and the suspected diagnosis may argue for a shorter or longer interval, and that this figure has never been tested in an HCC cohort. Listing status remains with the transplant team while the uncertainty persists. The proposed approach is set out in Figure 10 and Table 2.

The Fleischner Society recommendations were written for incidentally detected nodules and explicitly exclude patients with known cancer [32]; likewise, liver-specific non-invasive diagnostic criteria were developed for the cirrhotic liver, and the lung lies outside their validation. When extrahepatic deposits are assessed for treatment response, conventional size-based Response Evaluation Criteria in Solid Tumors (RECIST) apply, because the enhancement-based component of modified RECIST (mRECIST) was designed for viable intrahepatic HCC [33,34]. Therefore, apply each criterion only in the setting in which it was validated.

### 3.3. Functional Imaging and Uncommon Patterns

The role of functional imaging depends on the endpoint under consideration. A meta-analysis of 11 studies and 572 patients reported an FDG-PET sensitivity of 64% and a specificity of 95% for extrahepatic metastases and local residual or recurrent HCC taken together, an endpoint that pools several sites and leaves the pulmonary nodule unmeasured [35]. Dual-tracer FDG/11C-acetate PET/CT contributes to selected whole-body staging questions [36]. In a study of 104 patients undergoing PET/MRI, every patient with metastases was identified, yet only seven of 16 individual pulmonary metastases (44%) were detected, compared with all 16 on CT [37]; strong patient-level performance and a poor lesion-level yield in the lung can thus coexist within the same examination.

These techniques are most useful when conventional assessment leaves a management-changing question open, as they may reveal disease elsewhere or identify a more accessible biopsy target [36,37]; their availability, protocol and performance in the relevant population determine whether they are worth requesting. For a 6 mm pulmonary nodule, however, they rarely add information, and the absence of uptake in such a nodule leaves the question of benignity unresolved [2,31,37].

Cavitary, micronodular and other atypical metastatic patterns are described in the broader thoracic oncology literature, although their frequency and specificity in pure HCC remain poorly established [28,29]. When such a pattern is encountered, infection, a second primary tumour and treatment-related change remain in the differential diagnosis. The frequently cited report of pulmonary lymphangitic carcinomatosis, for instance, concerned a combined hepatocellular-cholangiocarcinoma, a different disease than pure HCC [38]. Reports of this kind widen the differential without establishing an HCC-specific pattern, which would require a dedicated series.

### 3.4. Airway Metastases and Haemoptysis

Endobronchial or peribronchial HCC metastases have been reported after apparently controlled hepatic disease and in patients with bilateral thoracic involvement [17,39], and haemoptysis, cough, wheeze or recurrent post-obstructive infection may be the presenting complaint. CT may show enhancing intraluminal tissue or a peribronchial mass with bronchial narrowing, distal mucus impaction, atelectasis or consolidation, appearances that are shared with primary lung cancer and with other endobronchial lesions, so that bronchoscopy is usually required to establish the diagnosis. Because these lesions are vascular and friable, anticipate bleeding and plan for it before attempting any biopsy [17,39].

One HCC-specific retrospective series supports bronchial artery embolisation (BAE) for haemoptysis associated with pulmonary metastases, with technical success in all 18 patients, clinical success in 17 (94%) and a hypervascular tumour blush on angiography in every case [40]. BAE is therefore a haemostatic option in selected patients, although the evidence rests on a small uncontrolled series, and neither comparative efficacy nor an antitumour effect has been demonstrated. Contrast-enhanced CT places the bleeding lesion in relation to the airway, the pleura, and the bronchial or other systemic vessels, and this anatomical map underpins the interventional radiologist’s procedural planning (Figure 2).

## 4. Vascular Extension, Pulmonary Tumour Embolism and Microangiopathy

### 4.1. The Hepatocaval Route

Macrovascular invasion is a marker of aggressive HCC, and the route it follows determines whether the thorax becomes involved [9,10]. Portal venous tumour thrombus remains within the portal venous system, whereas tumour extending through the hepatic veins reaches the IVC and the right atrium, where it may obstruct venous return, interfere with cardiac inflow, or embolise to the pulmonary arteries [10,21,41]. Obstruction of hepatic venous outflow may also be accompanied by dilated venous collaterals, such as the inferior phrenic vein (Figure 3). A report should therefore state how far cranially the thrombus extends, whether it is continuous, and include the right atrium whenever the hepatocaval axis is involved. Echocardiography adds information on mobility, intracardiac extension, and haemodynamic effect that cross-sectional imaging provides poorly or not at all [21].

### 4.2. CT and MRI of Tumour Versus Bland Thrombus

Enhancing soft tissue within a vein, an expanded vessel, intrathrombotic vessels and continuity with a hepatic tumour all point to tumour thrombus on multiphasic CT or MRI [9,42,43]. The A-VENA study combined four of these features with an AFP above 1000 ng/mL in liver-transplant candidates with portal vein thrombosis. Among 467 listed patients, 59 had portal vein thrombosis, and 12 had tumour thrombus; the presence of at least three of the five criteria identified tumour thrombus with 100% sensitivity and 93.6% specificity [42]. The derivation was, however, retrospective, single-centre and confined to the portal vein; along the hepatic veins, the IVC, the right atrium and the pulmonary arteries, the same features remain informative, but their diagnostic performance has not been tested.

MRI is most helpful when enhancement is equivocal or when the thrombus is intrinsically hyperintense on T1-weighted images, because dynamic post-contrast sequences and subtraction imaging demonstrate enhancement more clearly, provided that registration is adequate [44]. Diffusion-weighted imaging (DWI) and apparent diffusion coefficient (ADC) maps provide additional information, although thrombus composition and age produce considerable overlap. An early study suggested that diffusion measurements could distinguish malignant from bland portal thrombus [45], whereas a later study found no useful separation by ADC, with an area under the receiver-operating-characteristic curve of 0.52 [46]. Restricted diffusion should therefore be interpreted alongside enhancement, morphology and continuity, as one element of a pattern rather than as an independent criterion. Moreover, almost all of these data derive from portal vein thrombosis, and the hepatocaval axis has been studied far less.

Tumour and bland thrombus frequently coexist [43,46], so a report should identify the suspected enhancing tumour component and describe any associated non-enhancing thrombus separately, together with the degree of obstruction and the extent of involvement. Anticoagulation acts only on the bland component, and when both coexist, the treating team has to weigh the thrombotic against the bleeding risk in the individual patient [43]. Figure 3 follows the anatomical route and illustrates the distance between suspected and established tumour thrombus.

### 4.3. Pulmonary Tumour Embolism and Microangiopathy

Pulmonary tumour embolism (PTE) results from tumour material reaching the pulmonary arterial circulation. Although documented in HCC, much of the pathological and imaging literature pools several primary cancers [10,47]. Dyspnoea, hypoxaemia, chest pain or right-heart strain may closely mimic conventional pulmonary thromboembolism, and CT pulmonary angiography (CTPA) may show arterial filling defects, vascular enlargement or peripheral infarct-like opacities. Enhancement within the defect, or an accompanying hepatocaval tumour, raises suspicion when present, although both may be absent [43,47]. Microscopic embolisation, however, lies below the resolution of CT, so that a study negative for central emboli leaves tumour-related microvascular disease an open question.

Pulmonary tumour thrombotic microangiopathy (PTTM) combines microscopic tumour embolisation with thrombosis and fibrocellular intimal proliferation, culminating in pulmonary hypertension [48]. Most published cases involve gastric or other adenocarcinomas, and for HCC the evidence consists of a handful of isolated reports [22]; nevertheless, the combination of rapidly progressive dyspnoea, hypoxaemia and right-heart dysfunction, with a CTPA that shows no central embolus, should raise the possibility. Thin-section CT may show centrilobular ground-glass nodules, tree-in-bud opacities or septal thickening, findings that overlap with infection and other pulmonary vascular disorders [22,48].

Echocardiography assesses the right heart, and right-heart catheterisation characterises pulmonary haemodynamics where clinically feasible; however, demonstration of the characteristic vascular remodelling requires histology, which is often unsafe to obtain in a deteriorating patient. Pulmonary wedge aspiration cytology has been used to detect malignant cells and thus to support a diagnosis of tumour embolic disease, but cytology shows the cells without demonstrating the intimal proliferation that constitutes the histological substrate of PTTM [48]. The diagnosis therefore remains presumptive in most patients, and no HCC-specific diagnostic or therapeutic pathway has yet been studied prospectively.

## 5. Direct Transdiaphragmatic Extension and Diaphragmatic Metastasis

The hepatic dome lies in close contact with the diaphragm, allowing a peripheral HCC to extend contiguously, an event distinct from a separate metastatic deposit within the diaphragmatic muscle [49]; apparent involvement may, on histology, prove to be fibrous adhesion. In a surgical series of 52 patients undergoing combined hepatic and diaphragmatic resection, histology showed muscular invasion in 11 and fibrous adhesion in 41 [50]. Gross operative assessment thus overestimated invasion in roughly four of every five patients selected for en bloc resection; how often true invasion occurs in unselected HCC would require a different study design.

Multiplanar CT should assess the diaphragmatic contour, focal thickening or discontinuity, and any soft tissue crossing the hepatic–diaphragmatic interface [51,52] (Figure 4). Contact or loss of the fat plane raises suspicion, but invasion and adhesion produce the same appearance, particularly after treatment (Figure 5). MRI can depict the normally low-signal diaphragmatic band and clarify an equivocal interface, although interruption or altered signal within the band acquires meaning only in the clinical context [53]. A small cohort of recurrent HCC described infiltrative diaphragmatic thickening that progressed across serial examinations, arguing for comparing successive studies before assigning a cause [51].

When a local extension affects resectability, imaging should describe its extent and its relationships to the pleura, the lung and the adjacent vessels, leaving histological certainty to the pathologist [50,52,54]. En bloc resection has been reported in selected patients, including combined hepatic, diaphragmatic and pulmonary resection after treatment [55]; these reports demonstrate feasibility under particular conditions, whereas the decision depends on liver reserve, the total extent of the tumour and whether an adequate resection is achievable. Figure 4 shows the diaphragmatic interface alongside pleural disease and a cirrhotic mimic.

## 6. Pleural, Nodal, Pericardial and Chest-Wall Disease

### 6.1. Pleural Disease and the Role of Sampling

Pleural metastasis from HCC is documented mainly in case reports and small series, and its true prevalence remains unknown [14,15,20]. Both contiguous extension and metastatic dissemination are possible routes; name the route only when anatomy or pathology supports it. Pleural recurrence has been reported without visible residual intrahepatic tumour, so malignant pleural disease remains possible even when the liver appears controlled [14]. Dyspnoea or pleuritic pain may result from an effusion, whereas rupture of a hypervascular thoracic deposit may produce a haemothorax that requires immediate assessment [15].

Contrast-enhanced CT may show nodular pleural thickening, pleural masses, an effusion, or extension across the diaphragm or the chest wall [56] (Figure 6). Thoracic ultrasound characterises accessible fluid and guides sampling. These appearances overlap with those of other metastatic tumours, mesothelioma, and inflammatory pleural disease, so the hepatic origin of a pleural deposit ultimately rests on tissue [56,57]. MRI may clarify an uncertain local interface, and PET may identify another disease site or a biopsy target, although variable HCC avidity and inflammatory uptake keep its interpretation short of definitive [31,56].

Fluid analysis and cytology are part of the investigation, with the caveat that a negative result does not exclude malignancy: a meta-analysis of 36 studies and 6057 patients with malignant pleural effusions found an overall cytological sensitivity of 58.2% (95% confidence interval, 52.5–63.9%), which varied widely by primary tumour [58]. This estimate is derived from general pleural oncology and includes no HCC-specific figure. If concern persists after non-diagnostic fluid sampling, imaging, alternative diagnoses, and the question of pleural tissue should all be reconsidered.

Image-guided pleural biopsy and medical thoracoscopy are options when tissue is required, and the choice between them depends on disease distribution, procedural feasibility, and any effusion that must be managed at the same time [59,60,61]. Thoracoscopy offers direct inspection and targeted biopsies; however, it may still be non-diagnostic, and a negative procedure requires correlation with the subsequent clinical and imaging course. Because general comparative evidence does not favour one approach over the other, HCC management is decided case by case, weighing anticipated benefit against the patient’s condition and the target’s vascularity [61].

### 6.2. Nodes, Pericardium and Chest Wall

Review the mediastinal and cardiophrenic nodes together with the upper abdominal nodal stations. HCC imaging series document thoracic nodal metastases, and an isolated mediastinal metastasis has been reported [20,62,63]. Size and enhancement characterise the node, whereas its likely origin is inferred from previous studies, the distribution of disease, and other sites involved; these same elements determine whether sampling is needed, particularly when an isolated node would change the treatment intent. The anatomy on the current examination is more informative than any presumed sequence of lymphatic spread.

Pericardial disease should be distinguished from tumour extension into the right atrium via the IVC. A recent case report attributed a pericardial malignancy to HCC and documented one presentation, although its frequency and route remain unknown [18]. Echocardiography assesses effusion and haemodynamic compromise, CT defines the anatomical relationships (Figure 7), and cardiac MRI may assist with tissue characterisation [18,21]. In a patient with HCC and pericardial fluid or a mass, a second primary cancer, infection and non-malignant causes remain in the differential diagnosis until pathological and clinical evidence settles the attribution.

HCC can also produce osteolytic rib, sternal, or vertebral metastases, sometimes with an expansile, enhancing soft-tissue component [19,20,62]; CT shows cortical destruction and fracture, while MRI is better for marrow or spinal canal extension [20]. These lesions have to be distinguished from direct extension across the diaphragm and from procedure-related implantation along a documented access tract. Although hypervascularity supports suspicion in the appropriate context and matters for biopsy planning, it is shared with renal, thyroid, and other hypervascular tumours, so the hepatic origin ultimately rests on histology [19,20].

## 7. Thoracic Vascular Interfaces in Locoregional Treatment

Extrahepatic collateral supply is most relevant for peripheral and dome HCC treated with transarterial chemoembolisation (TACE). The inferior phrenic arteries are the most frequent feeders, while intercostal, internal thoracic and other systemic branches contribute depending on tumour location [11,13]. Recruitment occurs when the hepatic artery is patent and is driven by tumour size, contact with adjacent structures and prior treatment, the three features that indicate to the operator where to look [64]. Consequently, when a new basal thoracic finding appears after an intervention, the hepatic tumour’s arterial supply should be considered as part of the explanation.

Arterial-phase CT can depict an enlarged right inferior phrenic artery and its relationship to the tumour, whereas catheter angiography and cone-beam CT define the supplied territory during planning [11,65] (Figure 8). In a small study of 25 patients with malignant hepatic tumours, automated feeder detection on cone-beam CT identified 44 of 46 extrahepatic feeders, compared with 26 of 46 on non-selective angiography [66]; this result concerns vessel detection during a procedure in a selected cohort and has no bearing on metastatic spread. Pleural or pulmonary staining from inferior phrenic branches can mimic tumour blush and should be recognised before embolisation [67].

The same anatomy accounts for a number of complications, since embolisation may affect the diaphragm, the pleura or the adjacent lung, and a hypervascular thoracic deposit may bleed from recruited systemic vessels [11,15,68]. CT and angiography should separate tumour supply from non-tumour branches and relate the findings to the treated territory, bearing in mind that a collateral feeder is a vascular finding, whereas diaphragmatic invasion and thoracic metastasis are tissue diagnoses that each require their own evidence.

## 8. Thoracic Complications of HCC Treatment

Any new thoracic abnormality should be interpreted with the treatment record at hand, because the procedure, the target location, the arterial route, the embolic material or systemic agent, and the interval since treatment are what distinguish tumour progression from inflammatory, vascular or structural complications [11,69,70,71]. Timing narrows the differential diagnosis, but causality also requires that the imaging pattern and the clinical course agree with it. Figure 5 illustrates treatment-related complications together with their documented treatment intervals.

### 8.1. TACE, Pleuropulmonary Reaction and Lipiodol Embolism

Pleural effusion, basal atelectasis or a pulmonary opacity may follow treatment delivered through the inferior phrenic arteries [68]. A small early effusion without pleural nodularity is often reactive, and symptoms, evolution and competing explanations determine how far to investigate; in contrast, an enlarging effusion, respiratory deterioration or suspected infection warrants reassessment in its own right, whatever the timing suggests. No study has defined the time a reactive effusion takes to resolve, so persistence is judged clinically [11,68].

Pulmonary embolisation of iodised oil (Lipiodol) is an uncommon complication of conventional TACE, described in association with intratumoral arteriovenous shunting and extrahepatic collateral pathways [12,71,72]; large tumours, greater oil volumes, and vascular shunting have been associated with the risk, based mainly on retrospective evidence. Unenhanced CT may show high-attenuation branching or punctate pulmonary deposits with surrounding ground-glass opacity or consolidation, and these findings carry most weight in a patient who becomes dyspnoeic or hypoxaemic within hours or days of oil-based treatment [12,71].

The clinical spectrum extends from limited deposition through chemical pneumonitis to acute respiratory distress syndrome [71,72]. Because oil embolism produces no conventional filling defect, a contrast-enhanced CT read only for thromboembolism may miss it, and the unenhanced images carry the diagnosis; assessment then addresses the severity of respiratory failure and the alternative or coexisting causes, in particular, infection and pulmonary oedema, and severe cases may require intensive supportive respiratory care. The imaging finding and the clinical syndrome deserve separate description, since the amount of radiopaque material and the degree of physiological injury vary independently [12,71].

### 8.2. Pneumonitis After Yttrium-90 Radioembolisation

Transarterial radioembolisation with yttrium-90 microspheres may expose the lung when particles pass through hepatopulmonary shunts; therefore, pretreatment technetium-99m macroaggregated albumin imaging is used to estimate shunting and lung dose during planning, with technique, dosimetry, and applicable product-specific limits all contributing to the decision [73]. However, the estimate is imperfect, and both a systematic review and a Korean multicentre study document pneumonitis despite calculated doses that appeared acceptable [70,74].

In the Korean cohort, 732 patients with liver malignancies underwent both a lung-shunt study and follow-up; symptomatic radiation pneumonitis occurred in 13 (1.8%) and was fatal in six [74]. This figure reflects that particular cohort’s case mix and dosimetry, and a different centre may well observe a different rate. Symptoms appeared between one and seven months after treatment, with a median of about three months, as dyspnoea and cough accompanied by bilateral ground-glass opacity or consolidation (Figure 9). The diagnosis requires review of treatment timing and dosimetry, together with evaluation for infection, oedema and other drug-related injury [70,74], since a low planned dose lowers the probability without excluding the complication, and the CT pattern is shared with several of the alternatives.

### 8.3. Delayed Diaphragmatic Injury and Thoracobiliary Fistula

Thermal injury after ablation near the hepatic dome may leave a diaphragmatic defect and, sometimes years later, cause herniation of abdominal contents [75,76]; therefore, a new basal opacity or an air-containing structure warrants examination of coronal and sagittal CT reconstructions for bowel or liver crossing the diaphragm. Reported delays range from months to years and are too scattered across case reports to define a surveillance window. Suspected obstruction or strangulation requires urgent surgical assessment, and the reformatted images distinguish a structural hernia from metastatic disease before the axial appearance is misread as tumour [75,76].

Bronchobiliary and other thoracobiliary fistulas have been reported after ablation for HCC, particularly when a biloma or an infected collection develops near the diaphragm [77,78]. Bilioptysis is the strongest clue to a bronchobiliary communication, whereas fever, cough, right basal consolidation or a persistent pleural collection are far less specific. CT can show the hepatic collection, the diaphragmatic defect and the thoracic consequences, although the tract itself often remains invisible; in a retrospective HCC series of 11 patients, MRI showed an obvious fistulous tract in only two [78], so cholangiographic studies or dedicated biliary MRI may be needed to establish the communication. Published HCC cases describe percutaneous drainage and biliary decompression, with further intervention according to the anatomy and the persistence of the fistula; since all of this evidence comes from case reports and small retrospective series, the sequence of drainage, decompression and closure has to be chosen for the individual patient.

### 8.4. Systemic Therapy and New Pulmonary Opacities

Immune checkpoint inhibitors used in HCC may cause pneumonitis, including during atezolizumab plus bevacizumab treatment [79,80,81]. CT appearances include organising-pneumonia-like consolidation, ground-glass opacity, and, in severe injury, a diffuse alveolar damage pattern [69,82]. The radiological framework derives mainly from general oncology populations; HCC-specific reports, including fatal cases, establish that the complication occurs in this population, whereas its incidence and the diagnostic accuracy of imaging await cohort data [81]. Document the timing relative to immunotherapy, and consider infection, oedema, and tumour progression as alternative explanations.

Clinical assessment and respiratory severity guide the investigation, and bronchoscopy with bronchoalveolar lavage helps to exclude infection where feasible, although a severely ill patient may need corticosteroids before it can be performed [69]; attribution to an immune-related adverse event, like the decision on whether to continue treatment, rests on the clinical picture together with the imaging. Antiangiogenic therapy also makes bleeding history relevant, particularly when a hypervascular thoracic lesion is present; however, most evidence on bleeding risk in HCC describes gastrointestinal events, and the incidence and mechanism of thoracic haemorrhage with these agents remain undescribed [2,79].

## 9. Cirrhosis-Associated Thoracic Mimics

Hepatic hydrothorax is usually a predominantly right-sided transudative effusion related to portal hypertension and may develop without obvious ascites [83]. In a patient with HCC, the cirrhotic background makes it likely, yet each effusion still has to be assessed on its merits. New pleural nodularity, unexplained progression or atypical fluid findings should prompt a search for malignancy, infection and other causes. Thoracic ultrasound and fluid analysis complement CT, with the reservation that a smooth pleural surface and a transudative result are compatible with hydrothorax and also occur in malignant effusion [58,83].

Hepatopulmonary syndrome combines liver disease with abnormal arterial oxygenation and intrapulmonary vascular dilatation. Contrast echocardiography, together with assessment of arterial oxygenation, establishes the diagnosis, while CT may appear unremarkable [84,85]. Portopulmonary hypertension, in contrast, is pulmonary arterial hypertension arising in the setting of portal hypertension: echocardiography screens for it, and the diagnosis requires right-heart catheterisation together with the exclusion of alternative causes of pulmonary hypertension [86]. The updated International Liver Transplantation Society guidance separates true pulmonary vascular disease from high-output or volume-overload states. In HCC, rapidly progressive pulmonary hypertension should also raise the possibility of thromboembolic or tumour microvascular disease, as discussed in Section 4.3 [22,48,86].

## 10. Integrated Interpretation and Practical Decisions

Several mechanisms may operate simultaneously in the same patient: a dome tumour may recruit an inferior phrenic feeder and abut the diaphragm, while a pulmonary nodule or a pleural effusion may have an entirely separate cause [11,19,20]. A useful report therefore describes each finding, relates it to the hepatic disease and treatment, and states how confident the reader should be; Table 1 links the principal patterns to their clinical presentation, a focused differential diagnosis, and the next investigation.

**Table 1:** Thoracic findings in HCC: imaging, clinical context and a suggested next step. Actions require clinical assessment; evidence categories describe study design rather than formal certainty grades.

Mechanism	Principal Imaging Findings	Clinical Presentation	Main Pitfall	Suggested Next Step	Evidence
Pulmonary metastases (Section 3)	Solid nodules of variable size, often multiple and bilateral	Often incidental; cough or dyspnoea	Benign nodule, infection or second primary; negative FDG is not exclusionary	Compare thin-section CT; assess management consequences; apply Figure 10 if indeterminate	HCC cohorts and imaging series [5,6,19,27,30]
Airway metastasis (Section 3.4)	Endobronchial/peribronchial tissue: narrowing, atelectasis or post-obstructive opacity	Haemoptysis, wheeze or recurrent infection	Other airway tumours; bleeding with biopsy	Contrast CT; plan bronchoscopic sampling; consider arterial mapping/BAE for bleeding	HCC case reports and an 18-patient BAE series [17,39,40]
Hepatocaval tumour thrombus (Section 4)	Enhancing intravascular tissue; expansion and continuity towards IVC/right atrium	May be silent; venous congestion or cardiac symptoms	Coexisting bland thrombus; diffusion overlap	Multiphasic CT/MRI; echo for intracardiac extent or effect; identify components separately	HCC imaging studies; diagnostic data mainly from portal thrombosis [9,21,42,44,45,46]
PTE/PTTM (Section 4.3)	Arterial defects in macroscopic PTE; subtle centrilobular opacity/septal thickening with pulmonary hypertension in PTTM	Progressive dyspnoea, hypoxaemia and right-heart dysfunction	Negative central CTPA does not exclude microvascular disease	CTPA and echo; specialist haemodynamic assessment when feasible; cytology does not prove PTTM remodelling	Mixed-cancer pathology/review evidence and HCC cases [22,47,48]
Diaphragmatic disease (Section 5)	Thickening, discontinuity or transdiaphragmatic tissue	Often found on imaging; pleuritic or referred pain may occur	Contact and adhesion can mimic invasion	Multiplanar CT; selected MRI; pathological correlation if available	Selected HCC surgical cohorts and imaging series [50,51,53,54]
Pleura/nodes/pericardium/chest wall (Section 6)	Pleural nodularity/effusion; nodes; pericardial mass/fluid; destructive rib or soft-tissue lesion	Dyspnoea, pain, haemothorax or cardiac compromise	Cirrhotic, inflammatory or other malignant disease	US-guided fluid assessment or targeted tissue sampling; echo for pericardial disease; MRI for local extension as needed	HCC imaging series/cases; sampling data from general pleural oncology [14,15,18,20,58,61,63]
Treatment-related injury (Section 7 and Section 8)	Basal reaction; high-attenuation pulmonary oil; pneumonitis; hernia or fistula	Depends on treatment and interval; respiratory or infective symptoms	Progression, infection or oedema; timing alone is insufficient	Review treatment record and CT; assess severity; unenhanced CT for oil; biliary imaging for suspected fistula	HCC interventional cohorts, general pneumonitis guidance and structural-complication cases [68,69,71,74,75,76,77,78,81]
Cirrhosis-associated mimics (Section 9)	Effusion; vascular dilatation or signs of pulmonary hypertension, sometimes minimal CT change	Effusion, positional hypoxaemia or exertional dyspnoea	Assuming all findings arise from cirrhosis	Fluid analysis; contrast echo and oxygenation for HPS; right-heart catheterisation to confirm suspected PoPH	Guidelines and clinical reviews [83,84,85,86]

Abbreviations: BAE, bronchial artery embolisation; CT, computed tomography; CTPA, CT pulmonary angiography; echo, echocardiography; FDG, fluorodeoxyglucose; HPS, hepatopulmonary syndrome; IVC, inferior vena cava; MRI, magnetic resonance imaging; PoPH, portopulmonary hypertension; PTE, pulmonary tumour embolism; PTTM, pulmonary tumour thrombotic microangiopathy; US, ultrasound.

### 10.1. Chest CT Yield and Treatment Intent

Guideline recommendations for staging read differently once the diagnostic-yield setting behind them is known. Current American Association for the Study of Liver Diseases (AASLD) guidance includes chest CT in HCC staging [2]. In one retrospective study, however, only 135 of 780 treatment-naïve patients had been selected for chest CT, of whom 17 (12.6%) had pulmonary metastases, with multiple hepatic lesions and combined hepatic and portal venous invasion as independent predictors [25]. The 12.6% therefore represents the prevalence among patients referred for chest CT by their treating team, and the whole newly diagnosed population, most of whom were never scanned, will have a lower prevalence.

More extensive hepatic disease, macrovascular invasion and a raised AFP all increase the probability of a malignant thoracic finding [6,19,25,26], whereas limited intrahepatic disease in a selected transplant population is associated with a lower proportion of metastatic nodules [5]. The cost of missing a metastasis before transplantation or resection is nonetheless considerable, and curative intent raises that cost. It is the cost, far more than any change in prevalence, that justifies careful chest staging in these patients, and linking these two facts reconciles the practical importance of chest staging with the modest yield reported in selected early disease.

### 10.2. A Proposed Framework for Indeterminate Pulmonary Nodules

Figure 10 and Table 2 set out an author-proposed framework for the solitary or few indeterminate pulmonary nodules discussed in Section 3.2; the framework structures multidisciplinary discussion and has never been prospectively validated. The first question is whether clarifying the cause would change management at all, including the possibility of an infectious diagnosis or of a second primary tumour, after which the assessment draws on previous CT, morphology, growth and the hepatic context. A convincing benign explanation may justify surveillance, whereas uncertainty that survives this exercise brings sampling, and its likely value, into the discussion [5,30].

**Figure 10:**
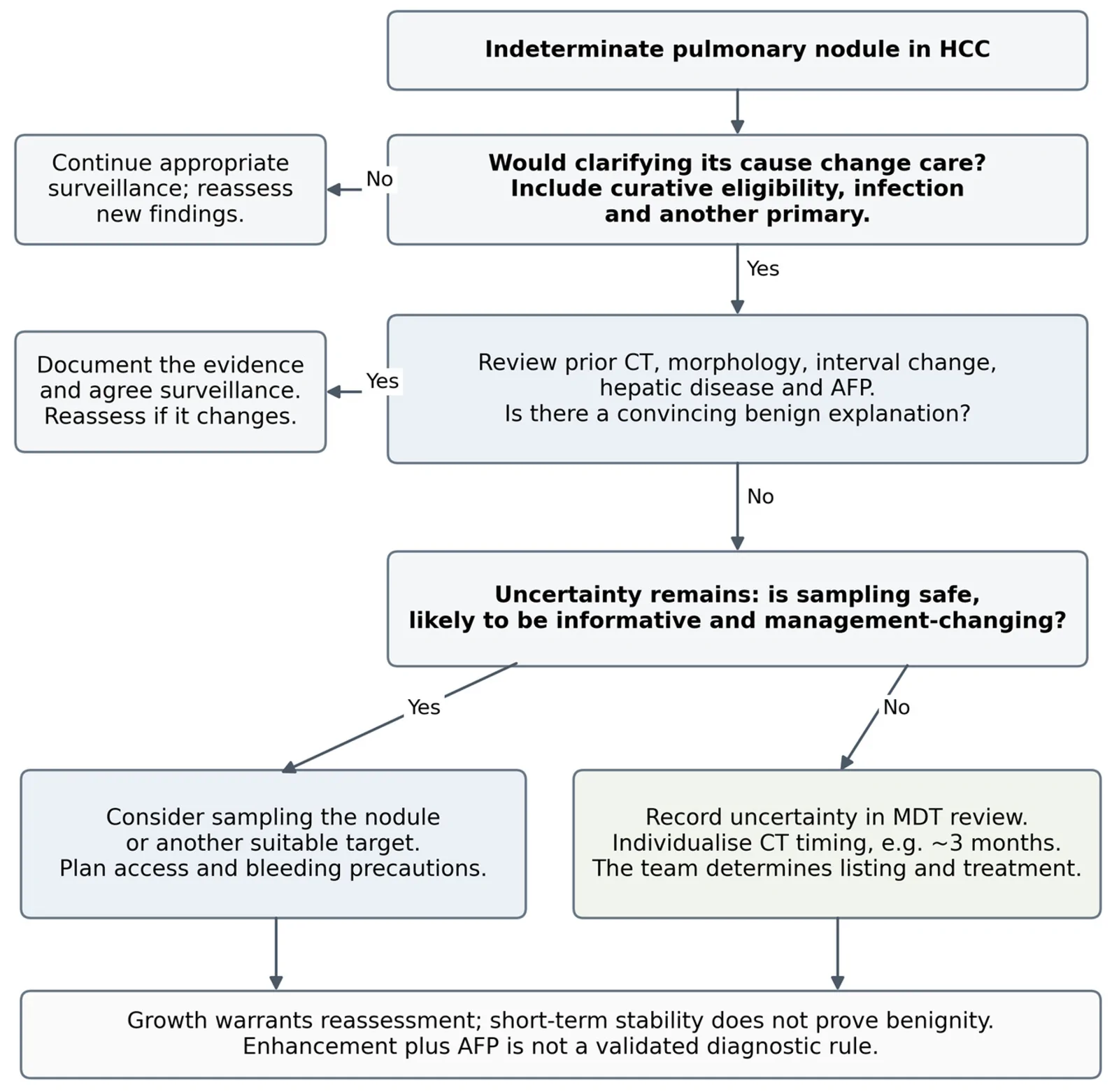
Author-proposed assessment of indeterminate pulmonary nodules during curative-intent HCC evaluation. The framework and illustrative follow-up interval are not validated HCC-specific rules. AFP, alpha-fetoprotein; CT, computed tomography; HCC, hepatocellular carcinoma; MDT, multidisciplinary team. A documented benign explanation can obviate surveillance; otherwise, uncertainty remains explicit during decisions about sampling and treatment.

Tissue confirmation deserves particular consideration when a new or enlarging lesion would close off an otherwise curative pathway and can be sampled safely [5], whereas in every other situation risk and expected yield weigh against it. When sampling is unlikely to help, the team should document the uncertainty and agree when to repeat CT and what the result would mean for listing or treatment. A hypervascular appearance with rising AFP raises suspicion and brings the case to the team sooner, but histology remains the gold standard [30]. Where several sites are available, the safer target is often the better one, and the illustrative three-month interval remains an individual decision.

**Table 2:** Practical application of the proposed framework in Figure 10.

Decision	Assessment	Suggested Action
Would the diagnosis change care?	Consider curative eligibility, infection and a second primary tumour.	If there is no actionable question, continue appropriate oncological surveillance; reassess if circumstances change.
Is there convincing benign evidence?	Review prior CT, morphology and duration of stability; interpret calcification by pattern.	Document the evidence and agree on surveillance. One short period of stability is insufficient.
Would sampling be safe and informative?	Consider size, access, vascularity, patient condition and another possible target.	Discuss tissue confirmation for management-changing uncertainty, especially a new or growing lesion.
Sampling has low expected value or disproportionate risk	No validated pulmonary enhancement/AFP rule removes uncertainty.	Record the uncertainty; individualise CT timing, e.g., about three months. The treating team determines listing and treatment.
The lesion grows or new findings develop	Reassess possibilities of metastasis, infection and another primary cancer.	Reconsider sampling or an alternative target; update the multidisciplinary decision.

### 10.3. Evidence Gaps and Research Priorities

This review is limited by targeted, non-exhaustive retrieval and by purposive selection. Rare manifestations are especially exposed to publication bias, and HCC treatment and imaging have changed enough over time to make comparisons across cohorts uncertain; moreover, diagnostic estimates drawn from selected transplant, surgical or interventional populations apply to those populations, and their transfer to unselected patients is an assumption. Evidence on general pleural sampling and on drug-related pneumonitis informs the investigation, while HCC-specific diagnostic accuracy for these methods remains to be measured [5,58,61,69], and the proposed nodule pathway awaits prospective evaluation.

Prospective HCC cohorts should record thoracic imaging findings alongside tumour stage, vascular invasion, treatment exposure, and the method used to establish each diagnosis; for pulmonary nodules, key endpoints include unnecessary exclusion from curative care, missed metastases, and the yield and consequences of sampling. Multicentre recording is also needed for endobronchial disease, PTE/PTTM, pleural and pericardial involvement, and post-treatment lung injury, and emerging radiomic models based on the hepatic tumour remain investigational until they have been independently validated for the clinical questions they are intended to answer [87,88].

## 11. Conclusions

Confirmed pulmonary HCC metastases usually reflect haematogenous dissemination. An indeterminate lung nodule, on the other hand, may be benign, infectious or a second primary malignancy. Thoracic abnormalities should therefore be interpreted in the light of the hepatic examination and the treatment record, with diagnostic confidence kept proportionate to the evidence behind it; CT carries the main assessment of the chest, while MRI, echocardiography, angiography and tissue sampling answer the specific questions that remain open.

The four-domain approach separates direct tumour involvement, vascular dissemination, treatment injury and cirrhosis-associated disease while allowing all four to coexist in one patient, and it supports a focused differential diagnosis and a sensible next investigation. The proposed nodule framework makes uncertainty explicit during curative-intent decisions, although non-invasive diagnosis of pulmonary metastasis remains beyond current criteria; a prospective study is needed to determine what these strategies achieve and what they cost.

## Figures and Tables

**Figure 1:**
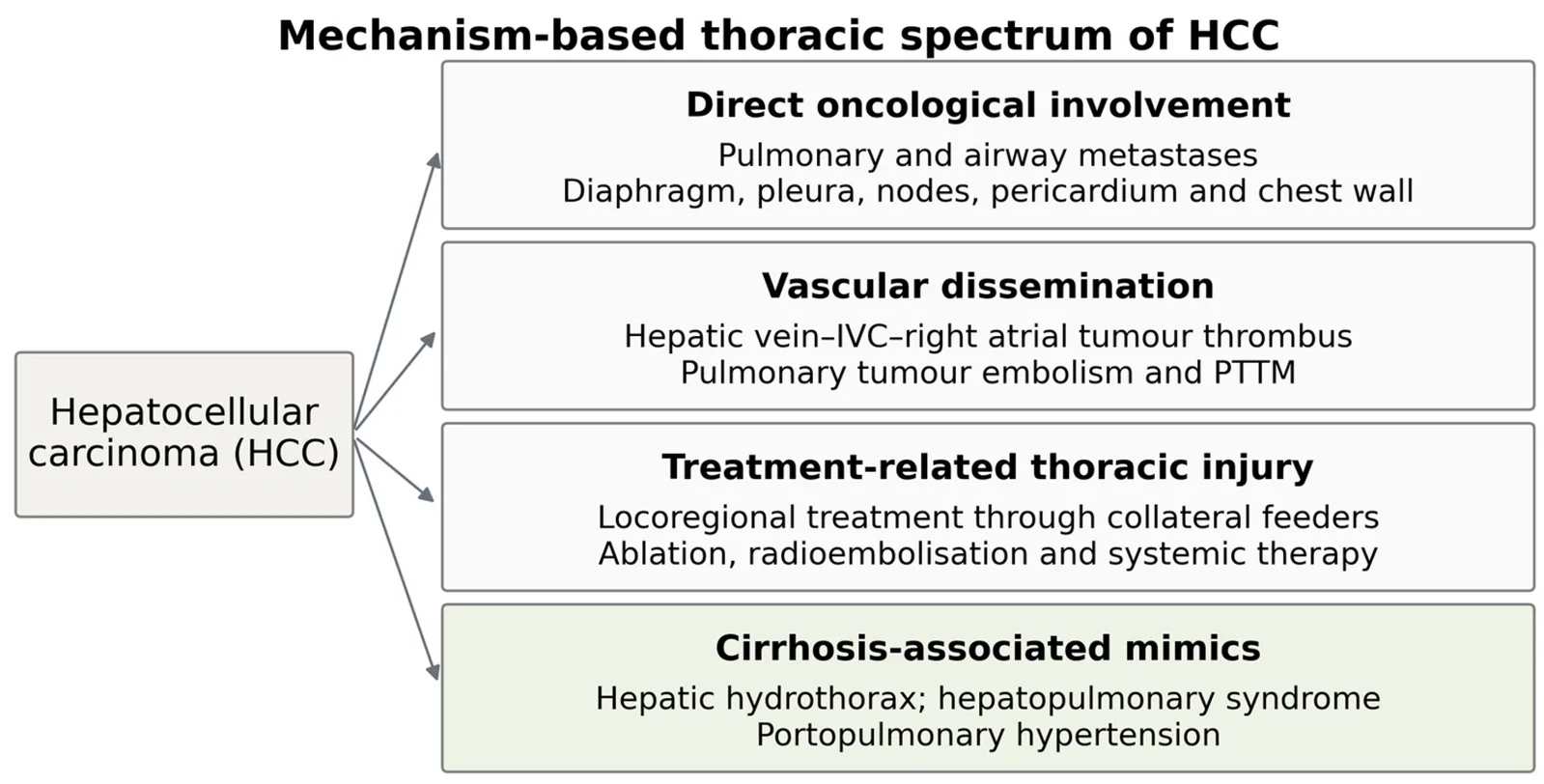
Four domains for interpreting thoracic findings in HCC. Collateral arterial supply is shown in relation to locoregional treatment and possible thoracic injury. IVC, inferior vena cava; PTTM, pulmonary tumour thrombotic microangiopathy.

**Figure 2:**
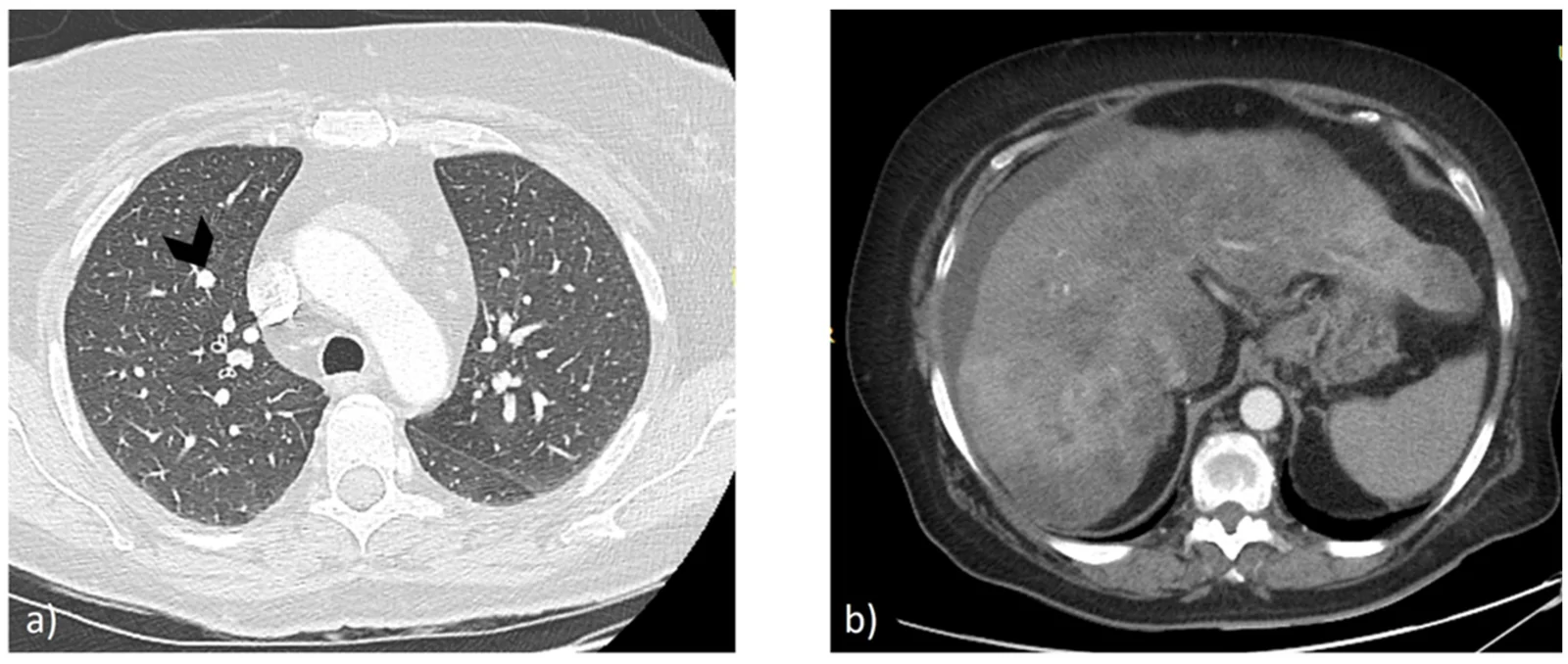
(**a**) Axial CT (lung window) identifies a 6 mm, non-specific, indeterminate pulmonary nodule in the apical segment of the right upper lobe, centrally located, with a regular contour and homogeneous, solid density (arrowhead). (**b**) Axial CT of the liver in the same patient shows diffuse infiltrative HCC and perihepatic free fluid.

**Figure 3:**
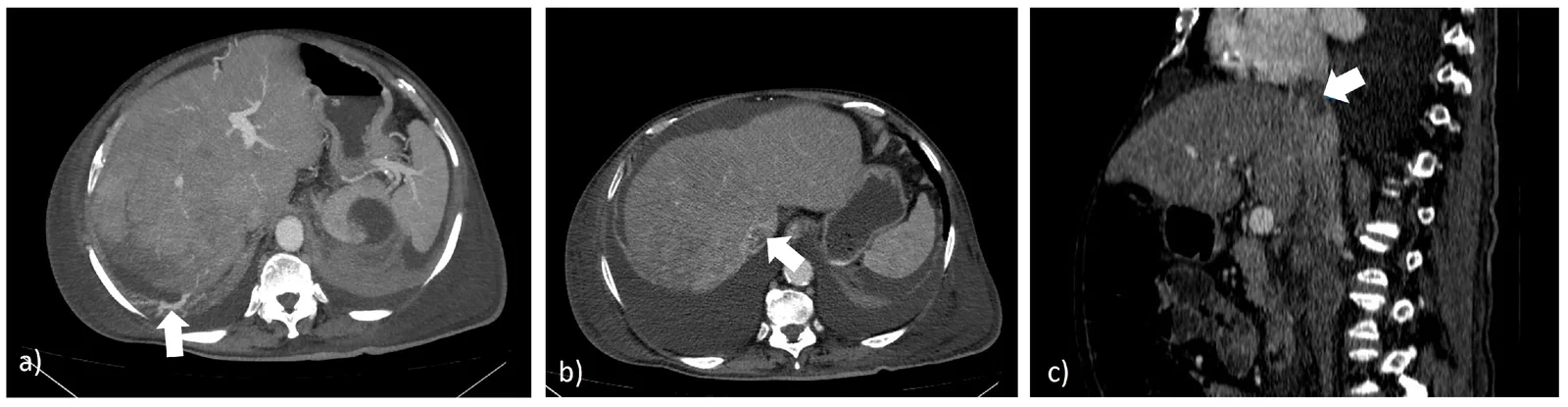
Contrast-enhanced computed tomography CT demonstrating tumoral venous thrombosis and an associated venous collateral pathway in a patient with hepatocellular carcinoma HCC. (**a**) Axial image shows a short, branching, enhancing tubular structure, at the bare area of the right hepatic lobe, coursing toward the diaphragmatic crus (arrow), consistent with a dilated right inferior phrenic vein functioning as a venous collateral in the setting of hepatic venous outflow obstruction. (**b**) Axial image at the level of the hepatic venous confluence demonstrates an expansile filling defect within the right hepatic vein extending into the inferior vena cava IVC (arrow), in keeping with tumoral thrombus. (**c**) Sagittal reformation confirms cephalad extension of the thrombus along the intrahepatic and suprahepatic inferior vena cava IVC (arrow).

**Figure 4:**
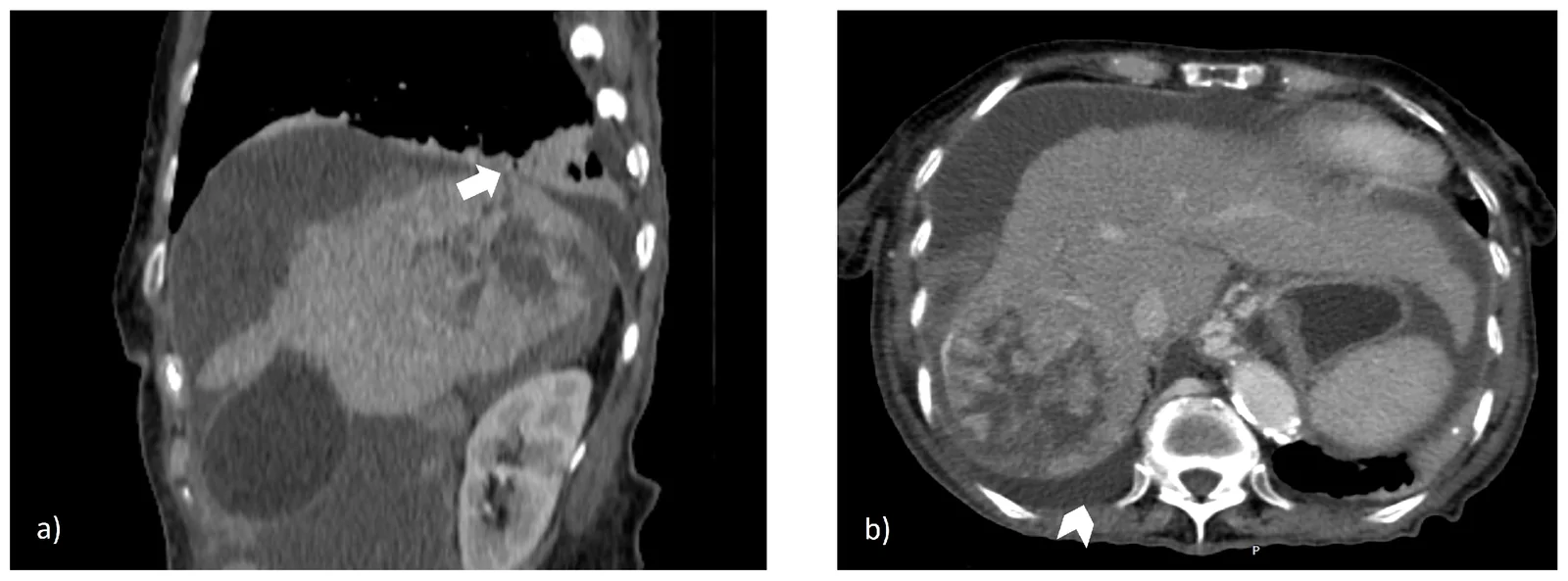
Contrast-enhanced computed tomography CT demonstrating a hepatocellular carcinoma HCC in hepatic segment VIII. (**a**) Sagittal reformatted image shows a heterogeneously enhancing mass occupying segment VIII, in direct contact with the right hemidiaphragm and effacing the normal hepato-diaphragmatic fat plane, diaphragmatic contour irregularity and focal discontinuity consistent with transdiaphragmatic tumour extension (arrow). (**b**) Axial image at the corresponding level confirms the segment VIII lesion abutting the diaphragm, with a small adjacent right pleural effusion (arrowhead), in keeping with transdiaphragmatic spread.

**Figure 5:**
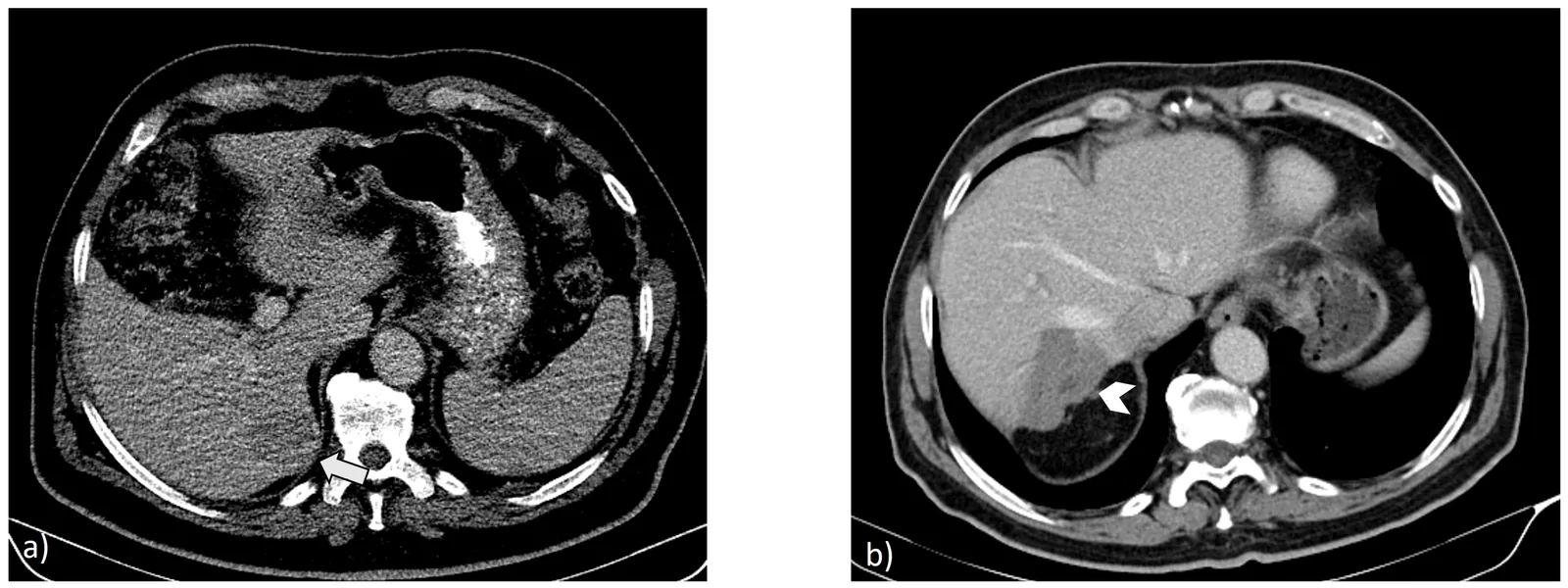
Contrast-enhanced CT of an HCC in hepatic segment VIII, before and after surgical ablation. (**a**) Preoperative image shows the tumour in intimate contact with the right diaphragmatic crus, raising a high index of suspicion for diaphragmatic invasion (arrow). (**b**) Follow-up image obtained four months after surgical ablation shows the ablation zone within the liver completely separated from the diaphragm (arrowhead). Together, the interval and postoperative appearance indicate that the preoperative contact reflected adherence rather than true invasion, since the lesion could be fully detached from the diaphragm at surgery.

**Figure 6:**
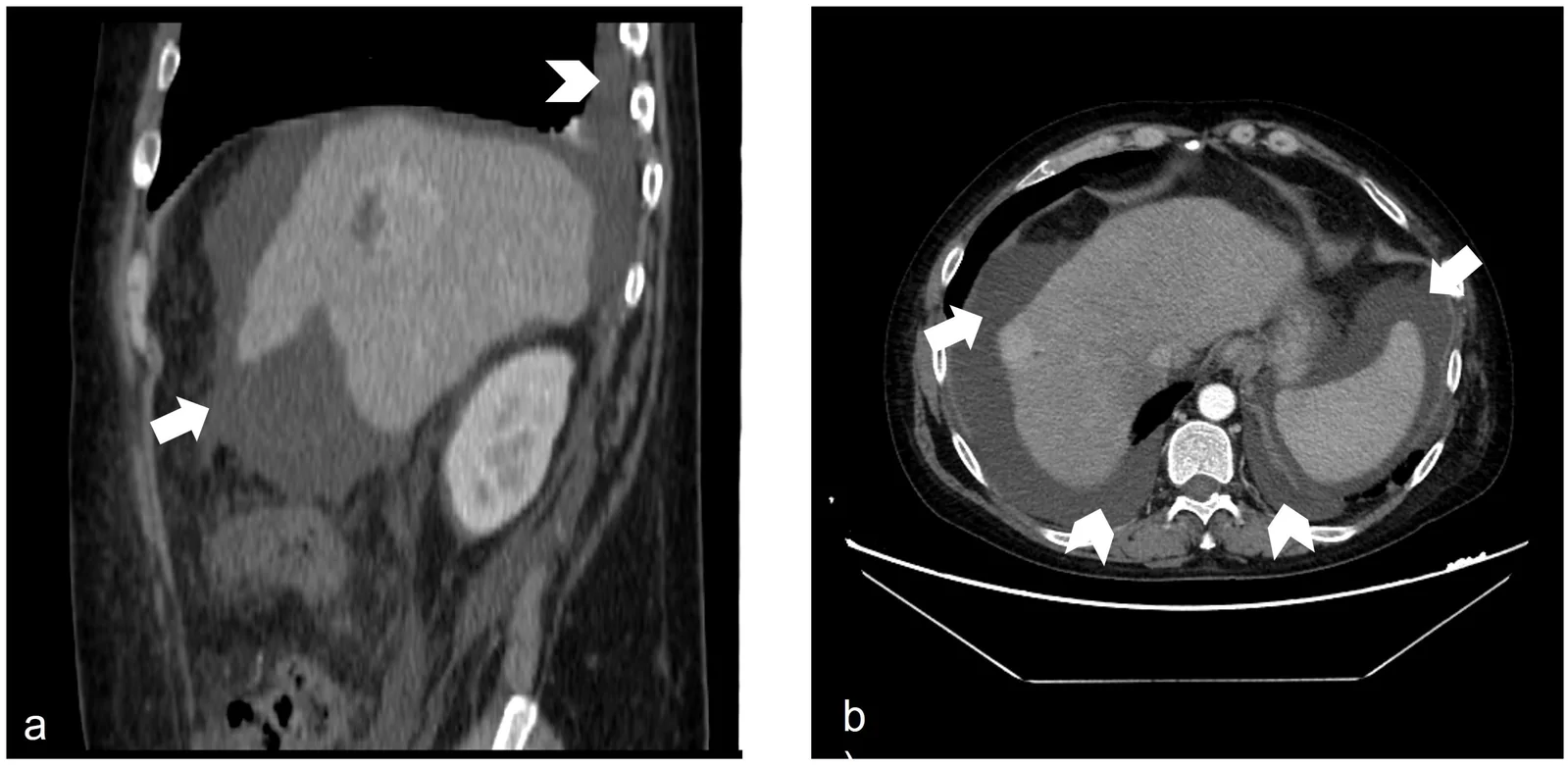
Contrast-enhanced computed tomography CT of the liver. (**a**) Right parasagittal multiplanar reformation demonstrates a hepatocellular carcinoma HCC within the hepatic parenchyma segment VIII. A thin, crescentic band of fluid attenuation outlines the anterior and inferior hepatic margin, consistent with perihepatic free fluid (arrow), and a small right-sided pleural effusion is present immediately superior to the right hemidiaphragm (arrowhead). (**b**) Axial image at the level of the hepatic dome confirms perihepatic and perisplenic free fluid outlining the margins of the liver and spleen (arrows), together with small bilateral pleural effusions (arrowheads).

**Figure 7:**
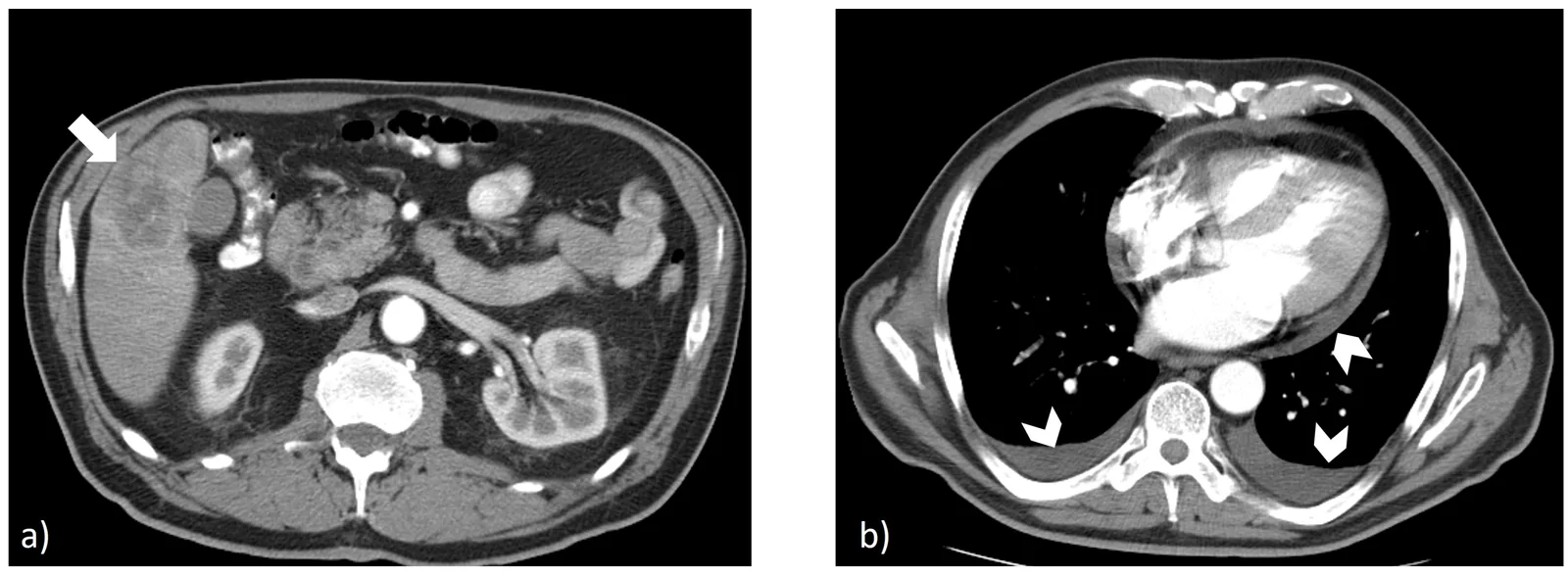
Contrast-enhanced CT in a patient with HCC. (**a**) Axial image at the level of the kidneys shows a subcapsular HCC in hepatic segment VI, along the postero-inferior margin of the right hepatic lobe (arrow). (**b**) Axial image at the level of the heart, obtained in the same patient, demonstrates coexistent pleural and pericardial effusion (arrowheads).

**Figure 8:**
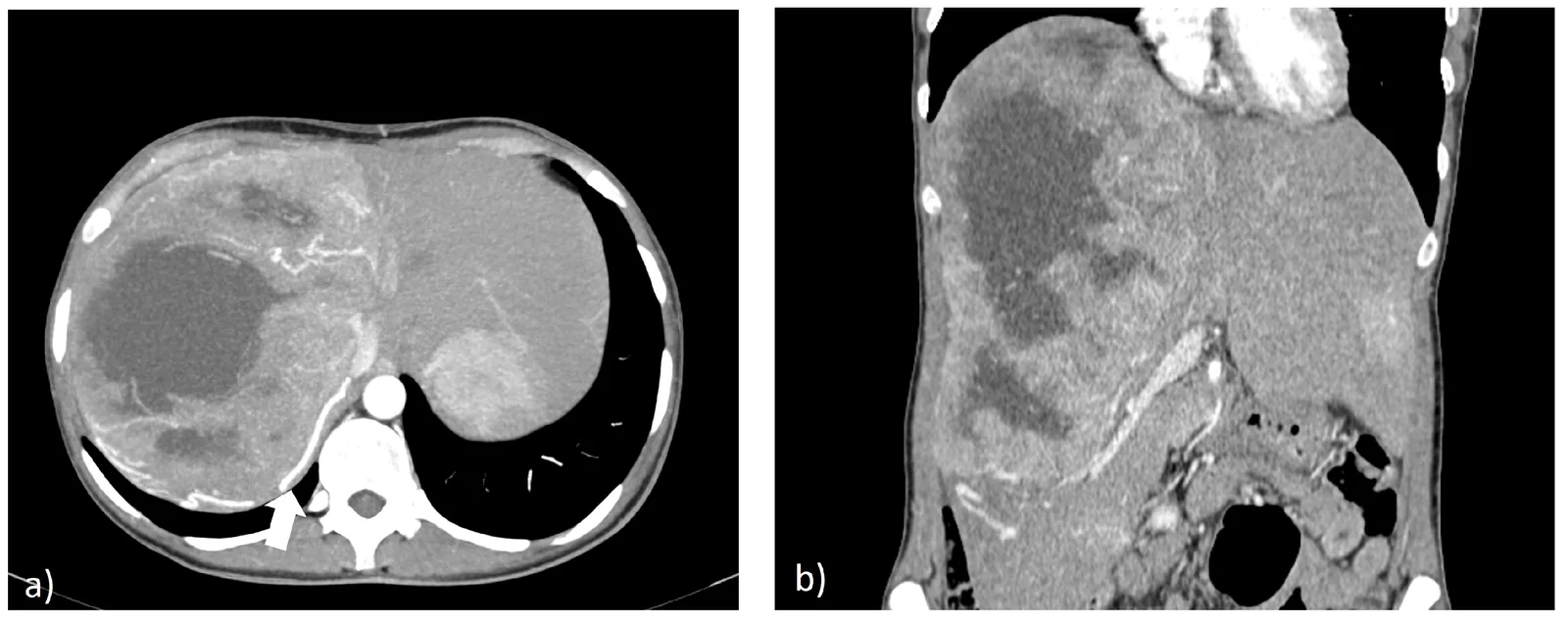
Contrast-enhanced computed tomography CT (thin-slab MIP reconstruction) of a large, necrotic hepatocellular carcinoma HCC. (**a**) Axial image shows a voluminous right hepatic mass with extensive central low-attenuation necrosis and an irregular, enhancing peripheral tumoral rim; a branch of the right inferior phrenic artery supplying this peripheral viable component is indicated (arrow). (**b**) Coronal reformation confirms the large size of the mass and its predominantly necrotic composition, with the corresponding peripheral enhancing rim.

**Figure 9:**
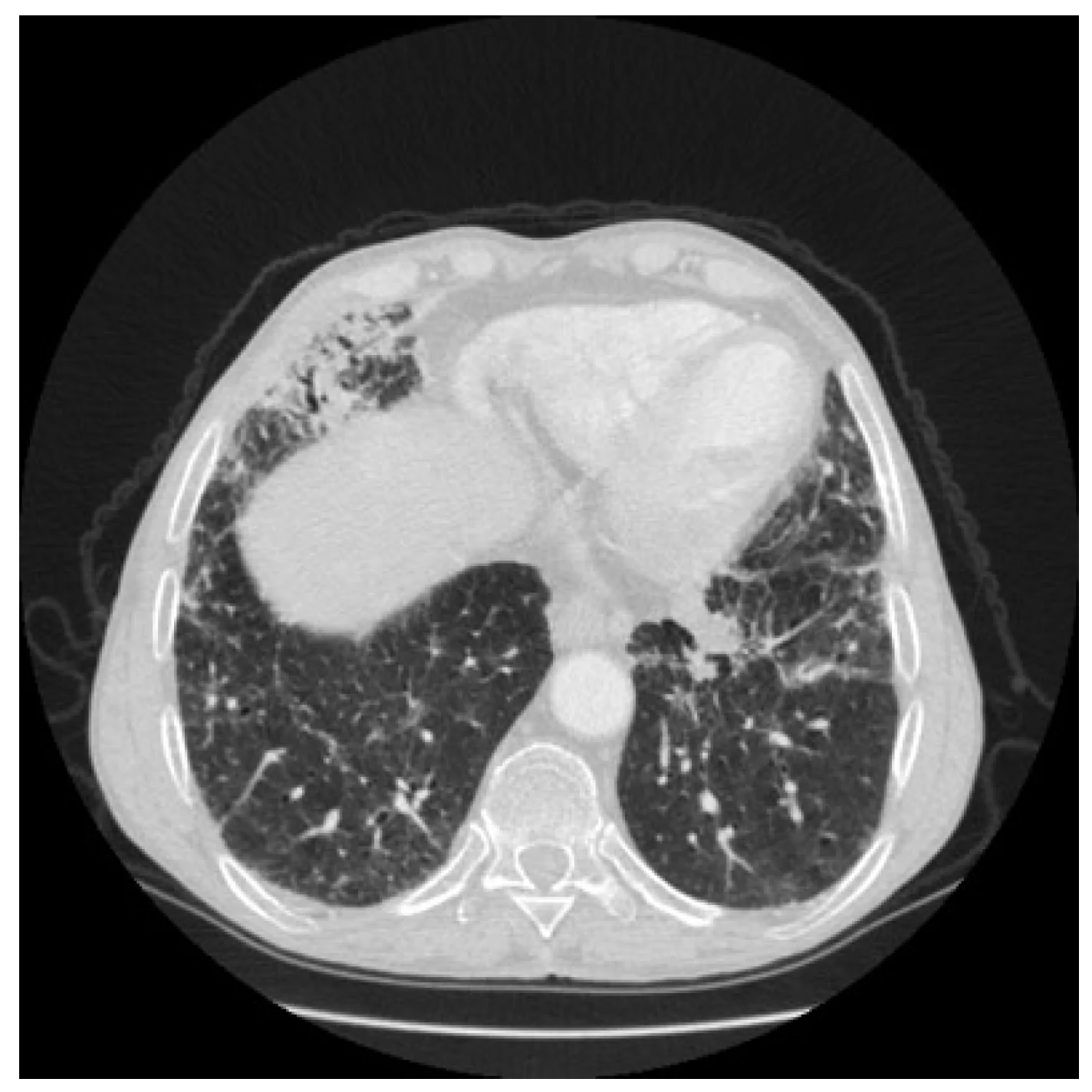
Axial CT (lung window) obtained several months after Yttrium-90 trans arterial radioembolization for hepatocellular carcinoma HCC shows diffuse, bilateral ground-glass opacities with superimposed fine reticular changes, both without a lobar or segmental distribution, consistent with radiation-induced pneumonitis.

## Data Availability

No new data were created or analyzed in this study. Data sharing is not applicable to this article.
